# A comparison of beliefs about exercise during pregnancy between Chinese and Australian pregnant women

**DOI:** 10.1186/s12884-015-0734-6

**Published:** 2015-12-22

**Authors:** Kym J. Guelfi, Chen Wang, James A. Dimmock, Ben Jackson, John P. Newnham, Huixia Yang

**Affiliations:** School of Sport Science, Exercise and Health, The University of Western Australia, 35 Stirling Highway, Crawley, Western Australia 6009 Australia; Department of Obstetrics and Gynecology, Peking University First Hospital, Xi’anmen Street No. 1, Beijing, 100034 China; School of Women’s and Infants’ Health, The University of Western Australia, 35 Stirling Highway, Crawley, Western Australia 6009 Australia

**Keywords:** Pregnancy, Exercise, Physical activity, Theory of Planned Behaviour

## Abstract

**Background:**

Despite the well-established benefits of exercise during pregnancy, many women remain inactive. This may be related, in part, to women’s beliefs about exercise in pregnancy, which are likely influenced by cultural background. Accordingly, the aim of this study was to compare attitudes, subjective norms, and perceived behavioural control toward exercise, together with current levels of exercise participation between Chinese and Australian women during pregnancy. A second aim was to determine the extent to which these factors predict intention to exercise within a Theory of Planned Behaviour framework.

**Methods:**

Pregnant women (22 ± 2 weeks of gestation) living in China (*n* = 240) and Australia (*n* = 215) completed a questionnaire designed to assess a) maternal beliefs regarding the importance of exercise in relation to other health behaviours, b) attitudes, subjective norms, perceived behavioural control and intentions toward exercise, and c) current levels of physical activity. One-way analyses of variance were used to compare the demographics, maternal beliefs, attitudes, subjective norms, perceived behavioural control, intentions to exercise, and current physical activity levels between the Chinese and Australian samples. Structural equation modelling was used to determine which factors predicted intention to exercise in the two samples.

**Results:**

Australian women reported higher levels of current exercise and intentions to exercise in the next four weeks of pregnancy compared with Chinese women. These observations were associated with higher instrumental attitudes, ratings of subjective norm, and perceived behavioural control toward exercise in the Australian women. Instrumental attitudes and perceived behavioural control predicted intention to exercise in the Australian women, while perceived behavioural control was the only predictor of intentions to exercise in the Chinese sample.

**Conclusions:**

Beliefs, attitudes, barriers and intentions towards exercise during pregnancy differ between cultures. Understanding these differences may assist in the design of exercise interventions to maximise exercise adherence and lifelong physical activity patterns.

**Electronic supplementary material:**

The online version of this article (doi:10.1186/s12884-015-0734-6) contains supplementary material, which is available to authorized users.

## Background

In the past, pregnant women were discouraged from exercise; however, it is now well established that participation in regular exercise during pregnancy can have many health benefits for both the mother and her child [1, 2]. Despite these well-established benefits, many women remain inactive, or significantly reduce their exercise participation during pregnancy [3, 4]. One theory which has been used to understand exercise participation during pregnancy is the Theory of Planned Behaviour, a well-established framework for investigating the relationship between beliefs, intention, and actual exercise behaviour [5]. Intention to exercise has been found to be a significant predictor of exercise behaviour during pregnancy [4], and this in turn may be influenced by attitudes to exercise, subjective norms (perceived pressure from significant others) and perceived behavioural control (perceived ease or difficultly of performing regular exercise) [5]. For instance, in a sample of primarily Caucasian American women, attitude was the strongest predictor of intention to exercise, followed by perceived behavioural control [6]. These factors appear to be preceded by behavioural beliefs (perceived advantages) about exercise improving mood, energy and fitness, while common control beliefs (perceived impediments to exercise) include physical limitations, tiredness, and a lack of time [7]. However, cultural factors may also influence exercise beliefs, exercise intentions, and exercise participation during pregnancy [8]. To our knowledge, no studies have directly compared these factors across cultures.

In traditional Chinese culture, pregnancy is considered a vulnerable period that requires rest and recuperation, with many antenatal taboos [9], some of which may contrast with international guidelines on exercise in pregnancy. Two relevant taboos intended to avoid spontaneous miscarriage include “not walking too fast” and “not walking too often”, which have been reported to be adhered to by the majority of Chinese women [9]. In a recent study of 1,056 pregnant women in the third largest city in China, Tianjin, women expressed concern about the safety of exercise, with the most common reason given for not exercising during pregnancy being a fear of miscarriage [10]. Accordingly, only 11 % of pregnant Chinese women met the international recommended guidelines for physical activity during pregnancy [11]. Furthermore, 74 % of Chinese women reported reducing their physical activity as their pregnancy progressed [10].

In contrast, the main barriers to exercise in Australian women have been reported to be similar to those observed in the Caucasian American population studied by Symons Downs & Hausenblas [8]; feeling too busy, too tired, too unwell, and because exercise was too uncomfortable [12, 13]. All of these factors were rated as a greater impediment than exercise being ‘unsafe’ [12]. Correspondingly, physical activity levels in Australian women are estimated to be higher, with reports of 44 % [14] and 33 % [15] of women representative of the typical obstetric population meeting international physical activity guidelines for pregnancy.

Given the apparent differences in beliefs about exercise during pregnancy between cultures, the first aim of this study was to directly compare attitudes, subjective norms, perceived behavioural control and intentions towards exercise during pregnancy, together with current levels of physical activity, between a sample of Chinese and Australian women. Our second aim was to determine the extent to which these factors predict intention to exercise within a Theory of Planned Behaviour framework. Gaining a greater understanding of women’s beliefs about exercise during pregnancy may inform the design of exercise interventions for this population to maximise exercise adherence and lifelong physical activity patterns.

## Methods

Pregnant women between 18–26 weeks of gestation were recruited in Beijing, China (*n* = 240) and Perth, Australia (*n* = 215). All women were asked to complete a questionnaire designed to assess their attitudes, subjective norms, perceived behavioural control and intentions toward exercise during pregnancy, together with their current levels of physical activity. Women completed the questionnaire while waiting for their antenatal appointments at Peking University First Hospital (Beijing, China) or King Edward Memorial Hospital (Perth, Australia). A portion of the Australian sample (*n* = 93) were also recruited from the larger community and these women completed the questionnaire online. Efforts were made to ensure that both the Beijing and Perth samples were representative of the range of women living in these urban centres, with the Perth women recruited primarily from Western Australia’s main public obstetric hospital (overseeing ~6,000 births each year), as well as local community forums. Meanwhile, the Beijing women were recruited from Peking University First Hospital, which is a major government hospital in Beijing providing both general and VIP obstetric clinics (overseeing ~5,500 births each year). Efforts were also made to match the environmental conditions at the time of survey administration between countries to limit any potential effect on exercise participation rates. Accordingly, the Chinese sample completed the questionnaire in April and May 2014 (mean monthly temperature range 8–27 °C), while the Australian sample completed the questionnaire between April and September 2014 (mean monthly temperature range 8–26 °C). Participation was voluntary and the study was approved by the Institutional Ethics Committees (Ethics Committee of Peking University First Hospital; Western Australian Women and Newborn Heath Service Ethics Committee and The University of Western Australia Human Ethics Committee).

The questionnaire assessed demographic variables such as age, height, body mass, health status, household income, country of birth and the duration of time living in either China or Australia. The questionnaire involved three additional components; a) an assessment of maternal beliefs regarding the importance of exercise in relation to other health behaviours, b) an assessment of attitudes, subjective norms, perceived behavioural control and intentions toward exercise during pregnancy, and c) an assessment of current levels of physical activity Additional file 1. Regular exercise was defined in the questionnaire as ‘physical activities that make your heart beat faster than normal and increase your breathing, and which you do for at least 30 minutes at a time, 3 times per week’. All components of the questionnaire were developed in English, with translation to simplified Chinese using an independent company (Language Scientific, MA, USA). Briefly, this involved three native speakers of simplified Chinese with an appropriate technical background examining the translation and negotiating any differences in opinion. Following this, the questionnaire was administered to two bilingualists for further confirmation of the translation.

### Maternal beliefs about health behaviours during pregnancy

First, women completed an assessment of maternal beliefs regarding the importance of exercise in relation to other health behaviours (modified from [16]). Briefly, this involved women rating the importance of performing 11 different health behaviours in pregnancy (e.g. getting a good night’s sleep, not smoking, not drinking alcohol, eating healthy foods, not drinking coffee, resting and relaxing, not worrying too much, stopping work, not gaining too much weight, exercise regularly and having an active lifestyle) on a 5-point Likert scale ranging from 1 (not at all important) to 5 (very important).

### Maternal attitudes, perceived norms, behavioural control, and intentions towards exercise

Second, women completed an assessment of attitudes, perceived norms, behavioural control, and intentions towards exercise during pregnancy. This component of the questionnaire was based on the Theory of Planned Behaviour guidelines [5]. Again, regular exercise was defined as ‘physical activities that make your heart beat faster than normal and increase your breathing, and which you do for at least 30 minutes at a time, 3 times per week’. The attitude instrument included eight items reflecting affective (four items e.g., unenjoyable-enjoyable) and instrumental (four items e.g., harmful-beneficial) attitudes that were scored on a seven-point Likert scale preceded by the stem “Regular exercise at this stage of my pregnancy is….”. Subjective norms were assessed based on four items scored on a seven-point Likert scale; “other pregnant women like me exercise regularly at this stage of their pregnancy”, “other pregnant women who I admire have exercised regularly at this stage of their pregnancy”, “most people who are important to me think that I should exercise regularly at this stage of my pregnancy” and “most people whose opinion I value would approve of me exercising regularly at this stage of my pregnancy”. Perceived behavioural control was assessed by four items relating to confidence to exercise and control over ability to exercise; “I am confident that I can exercise regularly at this stage of my pregnancy”; “I am in control of whether I exercise regularly at this stage of my pregnancy”; “Whether or not I exercise regularly at this stage of my pregnancy is up to me”; “For me, to exercise regularly at this stage of my pregnancy would be (1 very difficult; 7 very easy). Intention to exercise was assessed with two items using a seven-point Likert scale ranging from 1 (not true at all) to 7 (very true); “I intend to exercise regularly in the next 4 weeks of my pregnancy”; “I will make an effort to exercise in the next 4 weeks of my pregnancy”). Additional items were included to assess women’s beliefs about the main barriers to exercise reported previously. Specifically, four items addressed the degree to which tiredness, a lack of time, difficulty moving and concerns about safety prevented women “from exercising regularly at this stage of my pregnancy” (1 strongly disagree; 7 strongly agree).

### Maternal physical activity levels

Finally, women completed an assessment of current levels of physical activity using the International Physical Activity Questionnaire (Short version) which was developed for cross-national monitoring of physical activity and has been shown to be valid and reliable in a number of different countries [17]. Briefly, this involves assessment of the frequency and duration of walking, moderate-intensity physical activities (excluding walking) and vigorous intensity physical activities. From this, the number of MET-minutes per week is calculated for each category of activity, and for all activities combined

### Data analysis

Women were excluded from the analysis if they had been living in the country of interest for < 2.5 years (*n* = 22 for Australia). In addition, women were excluded from the analysis if they reported a significant health condition associated with their pregnancy (*n* = 13 for China; *n* = 7 for Australia). One-way analyses of variance were used to compare the background characteristics, maternal beliefs about health behaviours and exercise during pregnancy, and physical activity levels between countries. In order to examine the relations between attitudes (both instrumental and affective), perceived behavioral control, subjective norms and intentions, two structural equation models were specified using M*plus* Version 7.11 [18]; these models were identical but were estimated separately for Australian and Chinese women. Missing data were handled using a full information maximum likelihood (FIML) method of estimation under the assumption that they were missing at random. We specified a single model for each country that included all measurement (i.e., indicator) and structural (i.e., predictive pathway) parameters, and used a maximum likelihood estimator with robust standard errors (MLR) that accounts for the biasing effects of non-normality. Instrumental attitudes, affective attitudes, and perceived behavioral control were each initially specified using four indicators per latent variable, while injunctive norms, descriptive norms, and intentions were each formed using two indicators. We specified age and baseline physical activity levels as covariates in the model to estimate predictive pathways for attitudes, perceived behavioral control, and norms in relation to intentions while controlling for these factors. In addition, we specified covariance pathways between latent attitude, perceived behavioral control, and norm variables. We considered a range of indices in order to determine overall model fit [19, 20], namely the chi-square goodness-of-fit index, comparative fit index (CFI), Tucker–Lewis index (TLI), standardized root mean square residual (SRMR), and root mean square error of approximation (RMSEA). For the CFI and TLI, values > 0.90 were considered to indicate adequate fit, and those > 0.95 were considered to indicate excellent fit. For the SRMR and RMSEA, values between 0.05 and 0.08 were indicative of adequate fit, and values < 0.05 were indicative of excellent fit.

## Results

### Sample characteristics

The background characteristics of the study participants are shown in Table 1. The Australian women were heavier and taller, and also slightly older than the Chinese sample (*p* < 0.05). All women in the Chinese sample were born in China and of Asian ethnicity. In contrast, 40 % of the Australian sample was born in a country outside of Australia, with 76 % identifying themselves as Caucasian, 16 % of Asian ethnicity, 1 % Indigenous Australian and 7 % as other ethnicities. Those women born outside of Australia had been living in Australia for 14 ± 11 yr. For the Australia sample, annual household income was widely distributed (4 % < $50,000AUD; 15 % $50,000-99,000 AUD; 29 % $100,000-149,000AUD; 27 % $150,000-199,000AUD; 11 % $200,000-249,000AUD; 14 % ≥ $250,000AUD). For the Chinese sample annual household income was 3 % 24,000-69,999CNY; 23 % 72,000-119,999CNY; 74 % ≥ 120,000CNY. The Australian women rated their levels of participation in regular physical activity before becoming pregnant significantly higher than the Chinese women on a 7 point Likert scale (Australian 5.0 ± 1.8; Chinese 3.6 ± 1.8; *p* < 0.001).

**Table 1:** Participant characteristics (mean ± SD)

	Australia (*n* = 186)	China (*n* = 227)
Age (yr)	32 ± 4	30 ± 3*
Gestation (wk)	21 ± 3	22 ± 1*
Experiencing first pregnancy (%)	54	70*
Current body mass (kg)	70 ± 13	63 ± 9*
Pre-pregnancy body mass (kg)	65 ± 12	57 ± 9*
Height (m)	1.67 ± 0.08	1.63 ± 0.05*
BMI (kg/m^2^)	25.3 ± 4.2	23.7 ± 3.1

*Indicates significant difference between countries (*p* < 0.05)

### Maternal beliefs about health behaviours during pregnancy

Maternal beliefs about health behaviours during pregnancy are shown in Table 2 (1 = not important at all; 5 = very important). There were no differences in women’s beliefs about the importance of not smoking, not gaining too much weight, and exercising regularly between the Chinese and Australian women. The Chinese women viewed getting a good night’s sleep, not drinking alcohol, eating healthy foods, not drinking coffee, resting and relaxing, not worrying too much, stopping work and having an active lifestyle during pregnancy as more important compared with the Australian women (*p* < 0.01).

**Table 2:** Maternal beliefs about the importance of health behaviours during pregnancy (mean ± SD Likert rating ranging from 1 [not at all important] to 5 [very important])

	Australia	China
Get a good night’s sleep	4.7 ± 0.6	4.8 ± 0.4*
Not smoke	4.9 ± 0.5	5.0 ± 0.1
Not drink alcohol	4.7 ± 0.7	5.0 ± 0.3*
Eat healthy foods	4.6 ± 0.6	4.8 ± 0.5*
Not drink coffee	3.1 ± 1.0	4.6 ± 0.7*
Rest and relax	4.2 ± 0.8	4.8 ± 0.5*
Not worry too much	4.2 ± 0.7	4.6 ± 0.6*
Stop working	1.7 ± 0.8	2.5 ± 1.1*
Not gain too much weight	4.1 ± 0.8	4.2 ± 0.8
Exercise regularly	4.3 ± 0.7	4.3 ± 0.8
Have an active lifestyle	4.2 ± 0.7	4.6 ± 0.7*

*Indicates significant difference between countries (*p* < 0.05)

### Maternal attitudes, perceived norms, behavioural control and intentions towards exercise

Maternal attitudes, perceived norms, behavioural control and intentions towards exercise in pregnancy are displayed in Table 3. The Chinese women reported significantly lower instrumental attitudes toward exercise; however, there was no difference in affective attitudes between countries. With respect to subjective norms and perceived behavioural control, ratings were significantly lower in the Chinese women. Likewise, intentions to exercise in the next 4 weeks of pregnancy were significantly lower in the Chinese cohort.

**Table 3:** Attitudes, perceived norms, behavioural control and intentions towards exercise in pregnancy in Australian and Chinese women (mean ± SD)

	Australia	China
Instrumental attitudes	5.9 ± 1.2	4.9 ± 1.5*
Affective attitudes	4.8 ± 1.4	4.9 ± 1.5
Subjective norms	5.2 ± 1.0	4.7 ± 1.5*
Perceived behavioural control	5.4 ± 1.1	4.7 ± 1.3*
Intentions to exercise	5.7 ± 1.4	4.9 ± 1.6*

*Indicates significant difference between countries (*p* < 0.05)

Table 4 shows women’s beliefs about the specific barriers to exercise. The Australian women rated a lack of time as a greater barrier than the Chinese women, while the Chinese women rated concerns about the safety of exercise as a greater barrier than the Australian women (*p* < 0.001). There were no differences in beliefs about tiredness or difficulty moving the body preventing regular exercise between countries.

**Table 4:** Barriers to exercise during pregnancy in Australian and Chinese women (mean ± SD)

	Australia	China
Too tired	3.9 ± 1.7	3.9 ± 1.7
No time	4.0 ± 2.0	2.8 ± 1.7*
Difficulty moving body	2.7 ± 1.5	2.5 ± 1.5
Concerns about the safety of exercise	2.4 ± 1.5	3.4 ± 1.9*

*Indicates significant difference between countries (*p* < 0.05)

### Physical activity levels

Physical activity levels based on the IPAQ are shown in Table 5. Australian women reported greater levels of vigorous and moderate-intensity physical activity compared with the Chinese women (*p* < 0.001), although there was a wide variance between women. However, the Chinese women reported more walking (*p* < 0.001). When the total MET-minutes per week were calculated for all activities combined, there was no significant difference between groups.

**Table 5:** Physical activity levels of Australian and Chinese women during pregnancy (mean ± SD)

	Australia	China
Vigorous exercise (MET-min/week)	397 ± 989	5 ± 64*
Moderate exercise (MET-min/week)	422 ± 672	125 ± 378*
Walking (MET-min/week)	934 ± 992	1944 ± 1291*
Total (MET-min/week)	1782 ± 1952	2074 ± 1378

*Indicates significant difference between countries (*p* < 0.05)

### Predictors of intention to exercise during pregnancy

Within the Chinese sample, and with the exception of a significant chi-square value, the data were a relatively good overall fit for a measurement and structural model as described in the data analysis section, χ^2^(156) = 231.36, p = 0 .0001, CFI = 0.98, TLI = 0.97, SRMR = 0.03, and RMSEA = 0.046 (90 % CI [0.033, 0.058]). In terms of the measurement portion of the model, all intended factor loadings were classified as ‘fair’ or better (i.e. > 0.45) according to Comrey and Lee’s [21] recommendations, and composite reliability estimates for all measures were in the range 0.80 to 0.97. The primary aim in this analysis was to examine the extent to which theory of planned behavior ‘predictors’ were related to participants’ intentions to exercise. While controlling for participant age, physical activity level, and pre-pregnancy BMI, we observed one significant predictive effect for perceived behavioural control in relation to intention to exercise (standardized estimate = 74, *SE* = 0.27, p = 0.007, 95 % CI 0.205, 1.281). That is, among the pregnant Chinese women, participants reported stronger intentions to exercise when they felt capable of managing their exercise participation. No significant pathways emerged for either of the attitudinal or normative variables. Collectively, the theory of planned behavior variables, along with covariates, accounted for approximately 66 % of the variance in intentions (R-square estimate = 0.664, *p* < 0.001).

When estimating the same model within the Australian sample, the data initially displayed evidence of inadequate overall fit for a measurement and structural model as described in the data analysis section, χ^2^(156) = 349.05, *p* < 0.001, CFI = 0.88, TLI = 0.85, SRMR = 0.060, and RMSEA = 0.082 (90 % CI [0.070, 0.093]). Examination of the measurement portion of the model revealed that this unacceptable fit was due to one poorly-loading item within the perceived behavioral control variable (i.e., < 0.45 [21]), and also due to two covariance pathways that should be estimated between indicators (one between normative indicators and one between attitudinal indicators). Having specified a modified model that accounted for these issues (by dropping the poorly-loading item, and allowing two covariance pathways), with the exception of the significant chi-square value, the data displayed a good fit to the model, χ^2^(135) = 216.48, *p* < 0.001, CFI = 0.95, TLI = 0.93, SRMR = 0.045, and RMSEA = 0.057 (90 % CI [0.042, 0.071]); all factor loadings were fair or better according to Comrey and Lee’s criteria [21], and composite reliability estimates were in the range 0.62 to 0.93. In terms of structural pathways (i.e., predictors of intentions), while controlling for participant age, physical activity level, and pre-pregnancy BMI, perceived behavioural control (standardized estimate = 56, *SE* = 0.13, *p* < 0.001, 95 % CI 0.310, 0.816) and instrumental attitudes (standardized estimate = 0.32, *SE* = 0.12, *p* = 0.006, 95 % CI 0.089, 0.543) both emerged as significant predictors of intentions. That is, pregnant Australian women reported stronger intentions to exercise when they felt capable of managing their exercise participation, and/or when they strongly valued the benefits of exercise. Within the Australian sample, the theory of planned behavior variables, along with covariates, accounted for approximately 51 % of the variance in intentions (R-square estimate = 0.507, *p* < 0.001). Of note, for comparison purposes, we also estimated a model for the Australia-based sample in which all participants who declared themselves to be of Asian ethnicity were excluded (remaining *n* = 157). The data were a marginal fit for a measurement and structural model that mirrored our final Australian model reported above, χ2(135) = 264.80, *p* < 0.001, CFI = 0.92, TLI = 0.88, SRMR = 0.049, and RMSEA = 0.078 (90 % CI [0.064, 0.092]), and the significance of the predictive pathways was unchanged (i.e., perceived behavioural control and instrumental attitudes remained the only significant predictors of intentions).

## Discussion

Despite the well-established benefits of regular exercise during pregnancy for both the woman and her offspring, many women do not meet the recommended guidelines for exercise in pregnancy. The low rates of exercise participation during pregnancy are related, at least in part, to attitudes to exercise, subjective norms and perceived behavioural control [4]. In turn, each of these factors is likely influenced by cultural background; however, to our knowledge, no studies have directly compared these factors between Asian and Western cultures. We have found significant differences in beliefs about the importance of a number of health behaviours during pregnancy between a sample of Chinese and Australian women. With respect to exercise, Australian women reported greater levels of moderate and vigorous physical activity (but similar overall physical activity levels when walking is included), as well as greater intentions to exercise in the next four weeks of pregnancy compared with Chinese women. These observations were associated with higher instrumental attitudes toward exercise, ratings of subjective norm, and perceived behavioural control in the Australian sample. However, there was no difference in affective attitudes to exercise between countries. Instrumental attitudes and perceived behavioural control predicted intention to exercise in the Australian women, while only perceived behavioural control had significant effects upon intentions to exercise in the Chinese sample. These findings suggest that interventions targeted at increasing exercise during pregnancy should consider the cultural background of the pregnant woman.

With respect to maternal beliefs about health behaviours during pregnancy, the Chinese women viewed getting a good night’s sleep, not drinking alcohol, eating healthy foods, not drinking coffee, resting and relaxing, not worrying too much, stopping work and having an active lifestyle during pregnancy as more important compared with the Australian women. These differences may reflect the overall greater attention and concern given to pregnancy in Chinese culture which may be related, at least in part, to the Family Planning Policy [10]. There were no differences in women’s beliefs about the importance of not smoking, not gaining too much weight and exercising regularly between countries. The latter may be considered surprising given the higher instrumental attitudes toward exercise, ratings of subjective norm, perceived behavioural control and intentions to exercise in the next four weeks of pregnancy in the Australian sample compared with the Chinese women. Alternatively, the similar ratings of the importance of exercising regularly during pregnancy between samples may be considered consistent with the similar overall levels of current physical activity reported by the two samples (based on the total MET-minutes per week from all activities combined). However, it was interesting to note that a higher rating of importance was attributed to the sedentary behaviours of sleeping and resting/relaxing compared with exercise and having an active lifestyle in the Chinese women, while for the Australian women; resting/relaxing appeared to be of comparable importance with exercise and having an active lifestyle. Regardless, all behaviours were viewed as important (scored > 4) except for stopping work (both samples) and not drinking coffee (Australian women only).

Despite similar ratings of the perceived importance of exercise during pregnancy between Chinese and Australian women, significant differences were noted in maternal attitudes, subjective norms, perceived behavioural control and intentions towards exercise between the cohorts. The Chinese women reported significantly lower instrumental attitudes toward exercise, yet there was no difference in affective attitudes (feelings of enjoyment/pleasantness) between countries. This suggests that Australian women perceive more benefits to be gained from regular exercise during pregnancy. Such benefits may include improving mood, energy and fitness [7]. The reasons for a difference between cultures are unclear, but may be related to a greater awareness/education about the benefits of regular exercise during pregnancy in Western cultures, compared with the traditional Chinese view of pregnancy as a vulnerable period that requires rest and recuperation [9], with some traditional antenatal taboos contrasting with international guidelines on exercise in pregnancy. Indeed, Zhang and colleagues [10] reported that only 8 % of women received advice about physical activity from a medical professional in their sample of 1056 pregnant women in Tianjin, China.

Subjective norms and perceived behavioural control were also rated significantly lower in Chinese women compared with the Australian sample. The lower subjective norms (the views of significant others) in the Chinese sample again likely reflects the traditional Chinese view of pregnancy as a time for rest and recuperation. With respect to perceived behavioural control, the Theory of Planned Behaviour suggests that control beliefs (barriers to exercise) are a major contributing factor. Accordingly, the lower perceived behavioural control over exercise in pregnancy in the Chinese women suggests differences in the types or strength of barriers to exercise between cultures. Our data supports this notion, with the Australian women rating a lack of time and feeling too tired as the greatest barriers to exercise. In contrast, feeling too tired and concerns about the safety of exercise were the greatest barriers in the Chinese women. When directly compared, a lack of time was a significantly greater barrier in Australian women compared with the Chinese women, while the Chinese women rated concerns about the safety of exercise as a greater barrier compared with the Australian sample. These findings are consistent with previous observations that the main barriers to exercise reported by Australian women are feeling too busy or too tired [12, 13]. Likewise, the most common reason given for not exercising during pregnancy in a Chinese sample was a fear of miscarriage [10], however, this is the first study to directly compare between cultures.

Given the higher instrumental attitudes, subjective norms, and perceived behavioural control toward exercise in the Australian women, it is not surprising that intentions to exercise in the next four weeks of pregnancy were significantly higher in the Australian cohort. Likewise, Australian pregnant women reported greater levels of current vigorous and moderate-intensity physical activity compared with the Chinese women. However, it was interesting to note that the Chinese women reported significantly more walking. In fact, when the total MET-minutes per week were calculated for all activities combined, there was no significant difference between groups. These results suggest that despite engaging in less ‘planned’ moderate-vigorous physical activity during pregnancy, Chinese women may have a more ‘active’ lifestyle in general. Although no previous research has compared physical activity levels during pregnancy between cultures, previous studies have reported that Chinese women (in general) have lower rates of overall physical inactivity compared with Australian women [22, 23] and that walking accounts for the majority of physical activity in Chinese [22]. This has been attributed to an infrastructure or culture that supports walking in the Chinese [22].

Instrumental attitudes and perceived behavioural control were found to be significant predictors of intention to exercise in the Australian women. Likewise, attitude was the strongest predictor of intention to exercise, followed by perceived behavioural control in a sample of primarily Caucasian American women [6]. In contrast, perceived behavioural control was the only predictor to display significant effects upon intentions to exercise in the Chinese sample. These findings suggest that more research is needed, perhaps extending beyond the classic Theory of Planned Behaviour constructs, to understand what drives intentions to exercise in specific populations and, in turn, implement targeted strategies to increase exercise participation during pregnancy. Regardless, the current results show that consideration of the cultural background of a woman is necessary to design effective exercise interventions in pregnancy.

Although this is the first study to directly compare maternal beliefs about exercise between cultures, it is important to acknowledge some limitations. First, while all women in the Chinese sample were born in China and of Asian ethnicity, 40 % of the Australian women were born in a country outside of Australia, with 16 % identifying their ethnicity as Asian. Although this reflects the multi-cultural population of Australia, there is research to suggest that many Asian migrants continue to observe traditional practices relating to pregnancy and birth to varying degrees [24, 25]. However, it is worth noting that excluding participants who declared themselves to be of Asian ethnicity from the analyses did not alter the significance of the predictive pathways in our sample. In addition, it was not possible to strictly match the samples for gravidity, with more women in the Australian sample experiencing their second or third pregnancy. It is possible that experiencing pregnancy for a second or third time may influence the degree of caution. This may be an area for future research. It should also be acknowledged that despite attempts to ensure that the Beijing and Perth samples were representative of the range of women living in these urban centres, it is possible that differences in factors that were not measured including education status and social position may have influenced the results. Finally, it should be acknowledged that the measure of physical activity behaviour in the present study was retrospective (assessment of the past 7 days). Future studies may seek to align further measures of behaviour with the time orientation of items relating to the assessment of intention (i.e. in the next four weeks of pregnancy).

## Conclusions

In summary, this study has shown that beliefs, attitudes, barriers and intentions towards exercise during pregnancy differ between cultures. These findings suggest that cultural sensitivity is necessary in designing effective exercise interventions aimed at maximising exercise adherence and lifelong physical activity patterns. Importantly, attitudes and intentions towards exercise during pregnancy can be altered [13]. Accordingly, future studies should seek to design and test interventions targeted at improving exercise participation based on these results.

## Additional file

Additional file 1:
**Questionnaire.** (DOCX 47 kb)
