# Supplementary material for: A comparison of beliefs about exercise during pregnancy between Chinese and Australian pregnant women
Source: BMC Pregnancy Childbirth. 2015 Dec 22;15:345. doi: 10.1186/s12884-015-0734-6 (PMC4689036; doi:10.1186/s12884-015-0734-6)
Supplement: Additional file 1: — Questionnaire. (DOCX 47 kb) [file 12884_2015_734_MOESM1_ESM.docx]

| How many times have you been pregnant?  *(please circle)* | 1st pregnancy  2nd pregnancy  3rd pregnancy  Other________ | Do you have any health problems with your pregnancy? | Yes / No  If yes, please detail: ________________  ________________ |
| --- | --- | --- | --- |
| How many weeks pregnant are you? | _____ weeks | What is your age? | _____ years |
| What is your height? | _____ cm | What is your weight?  Current  Pre-pregnancy | _____ kg  _____ kg |
| Were you born in Australia? | Yes / No | What is your ethnic origin:  *(please circle)* | Aboriginal  Asian  Caucasian Other_____________ |
| If you were not born in Australia, how long have you lived in Australia? | _____ years | What is your yearly household income?  *(please circle)* | < $50,000  $50,000 - $99,000  $100,000 - $149,000  $150,000 - $199,000  $200,000 - $249,000  ≥ $250,000 |

**How important do you think it is for a pregnant woman to….. *(please circle)***

|  | Not important at all |  |  |  | Very important |
| --- | --- | --- | --- | --- | --- |
| 1. get a good night’s sleep? | 1 | 2 | 3 | 4 | 5 |
| 1. not smoke? | 1 | 2 | 3 | 4 | 5 |
| 1. not drink alcohol? | 1 | 2 | 3 | 4 | 5 |
| 1. rest and relax? | 1 | 2 | 3 | 4 | 5 |
| 1. not worry too much? | 1 | 2 | 3 | 4 | 5 |
| 1. not gain too much weight? | 1 | 2 | 3 | 4 | 5 |
| 1. exercise regularly? | 1 | 2 | 3 | 4 | 5 |
| 1. eat healthy foods? | 1 | 2 | 3 | 4 | 5 |
| 1. not drink coffee? | 1 | 2 | 3 | 4 | 5 |
| 1. have an active lifestyle? | 1 | 2 | 3 | 4 | 5 |
| 1. stop working? | 1 | 2 | 3 | 4 | 5 |

**For the following questions, think of ‘regular exercise’ as physical activities that make your heart beat faster than normal and increase your breathing (some ‘huff and puff’), and which you do for at least 30 minutes at a time, 3 times per week. Some examples are brisk walking, cycling or swimming.** *(Please circle)*

| 1. Before I was pregnant, I exercised regularly:  Not true at all 1 2 3 4 5 6 7 Very true |
| --- |
| 2. Regular exercise at this stage of my pregnancy is:  Harmful 1 2 3 4 5 6 7 Beneficial  Undesirable 1 2 3 4 5 6 7 Desirable  Detrimental 1 2 3 4 5 6 7 Valuable  Risky 1 2 3 4 5 6 7 Safe  Unpleasant 1 2 3 4 5 6 7 Pleasant  Not enjoyable 1 2 3 4 5 6 7 Enjoyable  Uncomfortable 1 2 3 4 5 6 7 Comfortable  Boring 1 2 3 4 5 6 7 Interesting |
| 3. Most people who are important to me think that I should exercise regularly at this stage of my pregnancy:  Strongly disagree 1 2 3 4 5 6 7 Strongly agree |
| 4. Other pregnant women like me exercise regularly at this stage of their pregnancy:  Strongly disagree 1 2 3 4 5 6 7 Strongly agree |
| 5. Most people whose opinion I value would approve of me exercising regularly at this stage of my pregnancy:  Strongly disagree 1 2 3 4 5 6 7 Strongly agree |
| 6. Other pregnant women who I admire have exercised regularly at this stage of their pregnancy:  Strongly disagree 1 2 3 4 5 6 7 Strongly agree |

| 7. I am confident that I can exercise regularly at this stage of my pregnancy:  Not true at all 1 2 3 4 5 6 7 Very true |
| --- |
| 8. For me, to exercise regularly at this stage of my pregnancy would be:  Very difficult 1 2 3 4 5 6 7 Very easy |
| 9. I am in control of whether I exercise regularly at this stage of my pregnancy:  Not true at all 1 2 3 4 5 6 7 Very true |
| 10. Whether or not I exercise regularly at this stage of my pregnancy is up to me:  Strongly disagree 1 2 3 4 5 6 7 Strongly agree |
| 11. I will make an effort to exercise regularly in the next 4 weeks of my pregnancy:  Definitely not 1 2 3 4 5 6 7 Definitely will |
| 12. I intend to exercise regularly in the next 4 weeks of my pregnancy  Not true at all 1 2 3 4 5 6 7 Very True |
| 13. Tiredness prevents me from exercising regularly at this stage of my pregnancy:  Strongly disagree 1 2 3 4 5 6 7 Strongly agree |
| 14. A lack of time prevents me from exercising regularly at this stage of my pregnancy:  Strongly disagree 1 2 3 4 5 6 7 Strongly agree |
| 15. Difficulty moving my body prevents me from exercising regularly at this stage of my pregnancy:  Strongly disagree 1 2 3 4 5 6 7 Strongly agree |
| 16. Concerns about the safety of exercise prevent me from exercising regularly at this stage of my pregnancy:  Strongly disagree 1 2 3 4 5 6 7 Strongly agree |

### INTERNATIONAL PHYSICAL ACTIVITY QUESTIONNAIRE

Think about all the **vigorous** activities that you did in the **last 7 days**. **Vigorous** physical activities are activities that take hard physical effort and **make you breathe much harder than normal**. Think *only* about those physical activities that you did for at least 10 minutes at a time.

1. During the **last 7 days**, on how many days did you do **vigorous** physical activities like heavy lifting, aerobics, or fast bicycling?

_____ **days per week**

No vigorous physical activities ***Skip to question 3***

1. How much time did you usually spend doing **vigorous** physical activities on one of those days?

_____ **hours per day** _____ **minutes per day**

Don’t know/Not sure

Think about all the **moderate** activities that you did in the **last 7 days**. **Moderate** activities refer to activities that take moderate physical effort and **make you breathe a little harder than normal**. Think only about those physical activities that you did for at least 10 minutes at a time.

1. During the **last 7 days**, on how many days did you do **moderate** physical activities like carrying light loads, bicycling at a regular pace, or doubles tennis? Do not include walking.

_____ **days per week**

No moderate physical activities ***Skip to question 5***

1. How much time did you usually spend doing **moderate** physical activities on one of those days?

_____ **hours per day** _____ **minutes per day**

Don’t know/Not sure

Think about the time you spent **walking** in the **last 7 days**. This includes at work and at home, walking to travel from place to place, and any other walking that you might do solely for recreation, sport, exercise, or leisure.

5. During the **last 7 days**, on how many days did you **walk** for at least 10 minutes at a time?

_____ **days per week**

No walking ***Skip to question 7***

1. How much time did you usually spend **walking** on one of those days?

_____ **hours per day** _____ **minutes per day**

Don’t know/Not sure

The last question is about the time you spent **sitting** on weekdays during the **last 7 days**. Include time spent at work, at home, while doing course work and during leisure time. This may include time spent sitting at a desk, visiting friends, reading, or sitting or lying down to watch television.

1. During the **last 7 days**, how much time did you spend **sitting** on a **week day**?

_____ **hours per day** _____ **minutes per day**

Don’t know/Not sure
